# HER2 evaluation in uterine serous carcinoma: diagnostic agreement between biopsy and resection samples

**DOI:** 10.1186/s12905-025-04235-8

**Published:** 2025-12-26

**Authors:** Zeynep Bayramoglu, Denizhan Bayramoglu, Safiye Aktas

**Affiliations:** 1 Department of Pathology, Dokuz Eylül University Faculty of Medicine Hospital, Izmir, Türkiye; 2 Department of Molecular Pathology, Institute of Oncology, Dokuz Eylül University, Izmir, Türkiye; 3 Izmır Sehir Hospital Department of Obstetrics and Gynaecology, Division of Gynaecological Oncology, Izmir, Türkiye

**Keywords:** Uterine serous carcinoma, HER2, Immunohistochemistry, CISH, Gene amplification, Biopsy, Concordance, Targeted therapy.

## Abstract

**Background:**

Uterine serous carcinoma (USC) is an aggressive subtype of endometrial cancer with high recurrence and mortality rates. HER2 overexpression and amplification represent important prognostic and predictive biomarkers in USC, supporting the use of HER2-targeted therapy. However, intratumoral heterogeneity and discrepancies between biopsy and hysterectomy specimens complicate accurate HER2 assessment. This study aimed to evaluate the diagnostic concordance of HER2 status between endometrial biopsy and resection samples using immunohistochemistry (IHC) and chromogenic in situ hybridization (CISH).

**Methods:**

This retrospective study included 40 patients diagnosed with USC (2010–2020). HER2, ER, PR, and Ki-67 were evaluated by IHC, and HER2 amplification was determined by CISH in all cases. Concordance between biopsy and resection specimens was assessed using Cohen’s kappa coefficient, with resection CISH considered the gold standard. Associations between HER2 status and clinicopathologic variables were analyzed using chi-square and Mann–Whitney U tests.

**Results:**

HER2 gene amplification was identified in 20% of cases (8/40). Biopsy CISH correctly detected 7 of 8 amplified tumors, yielding a positive predictive value (PPV) of 100% and a negative predictive value (NPV) of 97%. Only one true discordant case was observed. Concordance between biopsy and resection HER2 status was excellent (κ = 0.918). HER2-positive tumors demonstrated significantly higher Ki-67 proliferation indices (median 77% vs. 67%, *p* < 0.001) and were more frequently associated with advanced FIGO stage (*p* = 0.045). No significant associations were found between HER2 status and ER, PR, or LVI.

**Conclusion:**

Biopsy-based HER2 testing shows excellent concordance with resection specimens and reliably predicts HER2 amplification in USC. The findings highlight the utility of preoperative biopsies for HER2 assessment and affirm the importance of CISH confirmation in borderline (1 + and 2+) IHC categories. Standardized HER2 testing criteria specific to USC remain essential to optimize patient selection for targeted therapy.

## Introduction

Uterine tumors have the greatest incidence and the second highest fatality rate among gynecologic malignancies in the United States. Uterine serous carcinomas (USCs) constitute about 3–10% of all endometrial cancer diagnoses, although they account for around 50% of recurrences and over 40% of fatalities associated to endometrial cancer [1–3]. Consequently, USCs significantly impact morbidity and mortality rates. Five-year survival rates vary between 18% and 55%, and standard treatment approaches—consisting of thorough surgical staging followed by platinum-based chemotherapy—frequently prove inadequate, especially in cases of severe or recurring cancer. This highlights an important clinical necessity for the creation of innovative and more efficacious therapy strategies [3, 4].

In recent years, human epidermal growth factor receptor 2 (HER2) has become a significant prognostic indicator and therapeutic target, especially in breast, gastric, and colorectal malignancies. HER2 is a transmembrane oncoprotein that is essential for cellular proliferation, survival, and growth signaling pathways. Data from clinical trials reported in 2018 validated the prognostic and predictive importance of HER2 in patients with advanced or recurrent USC [5]. The use of anti-HER2 targeted therapy with chemotherapy markedly enhanced both progression-free survival and overall survival in this patient population. Consequently, HER2 testing has swiftly evolved into a standard auxiliary test during diagnosis.

HER2 is a biomarker with both prognostic and predictive significance in USC. In recent years, the development of HER2-targeted therapies has further increased the clinical relevance of this marker. Studies demonstrating that trastuzumab and other HER2 inhibitors improve survival in patients with advanced or recurrent USC highlight the critical importance of accurate and reliable HER2 assessment in therapeutic decision-making. Therefore, precise determination of HER2 status in preoperative biopsy specimens is of great clinical value, as it enables the appropriate selection of patients who may benefit from targeted therapy [4, 5].

The extensive implementation of HER2 testing in USC has uncovered considerable diversity in reported HER2 positivity rates, primarily because to discrepancies in testing methodology, interpretation criteria, and scoring systems. HER2 positive has varied from 12.3% according to the 2007 ASCO/CAP breast cancer guidelines, to 16.3% under the 2018 guidelines, and decreased to 10.5% using next-generation sequencing (NGS) techniques [6, 7]. This diversity underscores the necessity for uniform testing methodologies to enhance patient selection. The evaluation of HER2 in USC poses distinct problems, including as tumor heterogeneity and particular staining patterns. Inconsistencies in HER2 status have been documented among biopsy specimens, hysterectomy samples, and metastatic lesions. Discordance between biopsy and hysterectomy specimens has been often seen.

European studies have demonstrated that HER2 overexpression and gene amplification play a significant role in the biological behavior of USC. A large multi-institutional investigation involving several European centers reported substantial inter-laboratory variability in the immunohistochemical assessment of HER2 in USC, emphasizing the need for a scoring system distinct from that used in breast carcinoma and tailored specifically to these tumors. This finding highlights the necessity for standardization of HER2 testing across Europe [8–10]. Furthermore, a large population-based cohort study from Finland by Aro et al. (*n* = 1,239) showed that HER2 gene amplification occurs only in a small subset of endometrial cancers, but is predominantly concentrated in p53-abnormal/serous-type tumors, underscoring that serous carcinomas represent a biologically unique subgroup with respect to HER2 status [9]. Additional European cohorts have similarly reported that approximately one-third of uterine serous papillary carcinomas exhibit HER2 overexpression and/or amplification, a pattern associated with more aggressive clinical behavior. Taken together, the European literature strongly supports the role of HER2 as both a prognostic and therapeutic biomarker in USC, reinforcing the importance of accurate HER2 evaluation in guiding clinical management [8–10].

This study aims to examine HER2 status in endometrial biopsy and hysterectomy materials from the same USC patients utilizing immunohistochemistry (IHC) and in situ hybridization (ISH) techniques. Our objective is to address existing testing problems and to facilitate the creation of USC-specific standardized HER2 testing recommendations.

## Materials and methods

This retrospective analysis examined archival data of 40 patients diagnosed with USC at our institution from 2010 to 2020. The selection of cases in our study was performed meticulously to ensure a homogeneous biopsy cohort and to improve the reliability of the concordance analysis. Accordingly, only endometrial biopsies obtained exclusively by dilatation and curettage (D&C), along with the corresponding hysterectomy specimens from the same patients, were included. Patients who had undergone aspiration biopsy, Tru-Cut sampling, or hysteroscopic procedures; cases in which biopsy or hysterectomy materials had been submitted to our department as external consultations; and specimens with inadequate fixation quality in either the biopsy or hysterectomy samples were excluded. These exclusion criteria were implemented to minimize pre-analytical variability and to enable the most accurate assessment of HER2 concordance between biopsy and resection specimens.

All tissues included in the study were fixed according to standard protocols using 10% neutral buffered formalin. Similarly, hysterectomy specimens were delivered to the laboratory immediately after surgery without delay, sectioned appropriately, and placed in 10% neutral buffered formalin for fixation. To further minimize pre-analytical variation in HER2 immunohistochemical assessment, all hysterectomy specimens were subjected to a fixation period of at least 24 h. This standardized tissue fixation and processing approach was intentionally applied to prevent HER2 evaluation discrepancies between biopsy and resection samples from being influenced by pre-analytical factors.

Immunohistochemical (IHC) tests were done on sections derived from paraffin-embedded blocks of both endometrial biopsy and hysterectomy materials from the selected cases. The indicators evaluated in the IHC analysis comprised HER2, estrogen receptor (ER), progesterone receptor (PR), and Ki-67. HER2 expression was assessed according to the standards set by the American Society of Clinical Oncology/College of American Pathologists (ASCO/CAP) [9]. HER2 scoring was classified as 0, 1+, 2+, or 3+. All cases underwent chromogenic in situ hybridization (CISH) investigation to ascertain the HER2/CEP17 ratio.

Expression levels of ER and PR were assessed by the H-score method, determined by multiplying staining intensity (+ 1, + 2, +3) by the percentage of positively stained cells. Ki-67 was assessed as the proportion of positively stained nuclei. In our study, the assessment of lymphovascular space invasion (LVSI) was performed in accordance with the College of American Pathologists (CAP) “Protocol for the Examination of Specimens from Patients with Carcinoma of the Endometrium” (Version 5.1.0.0). Based on this guideline, only cases demonstrating five or more foci of LVSI were classified as LVSI-positive. This approach was implemented to identify unequivocal LVSI and to ensure a more homogeneous and clinically meaningful categorization of cases. Tumor staging was conducted in accordance with the FIGO classification system.

Statistical analyses were performed using Python 3.11 with the SciPy and Pandas libraries. Concordance between biopsy and resection HER2 status was evaluated using Cohen’s kappa coefficient, with resection CISH results serving as the diagnostic gold standard.

Associations between HER2 status and categorical clinicopathologic variables—including ER positivity, PR positivity, lymphovascular space invasion (LVI), and FIGO stage—were examined using the chi-square test; when expected cell counts were < 5, Fisher’s exact test was applied. Differences in non-normally distributed continuous variables (age and Ki-67 proliferation index) between HER2-positive and HER2-negative groups were assessed using the Mann–Whitney U test, and results were reported as median and interquartile range (IQR).

The diagnostic performance of biopsy-based HER2 CISH was determined through calculation of sensitivity, specificity, positive predictive value (PPV), and negative predictive value (NPV) using resection CISH as the reference standard. A p-value < 0.05 was considered statistically significant.

## Results

A total of 40 patients with uterine serous carcinoma were included in the study. HER2 amplification was detected in 20% of cases (8/40) based on resection CISH, which served as the diagnostic gold standard. Among the eight amplified cases, seven were correctly identified as positive in biopsy specimens, while one case was false-negative. Biopsy CISH correctly identified 32 of 33 non-amplified tumors, yielding a sensitivity of 87.5%, specificity of 100%, PPV of 100%, and NPV of 97%. Overall concordance between biopsy and resection HER2 status was excellent, with a Cohen’s kappa value of κ = 0.918, indicating *almost perfect agreement*.

Among the 40 paired biopsy–resection samples, six cases (15%) demonstrated an IHC score discrepancy between biopsy and hysterectomy specimens. All discrepancies represented minor (one-step) shifts such as 0→1+, 1+→2+, or 2+→1+ (Table 1). All tumors with an IHC score of 0 were CISH-negative, while CISH positivity was detected in one case with an IHC score of 1 + and in three cases with an IHC score of 2+. All IHC 3 + cases demonstrated HER2 gene amplification, confirming the strong association between high-level protein expression and underlying gene amplification.

**Table 1:** Comparison of HER2 immunohistochemical (IHC) scores and CISH results in biopsy and resection specimens. For each patient, the biopsy HER2 IHC score (0, 1+, 2+, 3+), biopsy CISH result, resection HER2 IHC score, and resection CISH result are listed individually. Cases labeled as “Amp” in the resection CISH column were considered true HER2-positive. This reference standard was used to evaluate discrepancies between biopsy- and resection-based HER2 assessments. (“Amp”: HER2 gene amplification; “0”: no amplification detected.)

Patient Number	Biopsy HER2 Score	Biopsy CISH	Resection HER2 Score	Resection CISH
1	+ 3	Amp	+ 3	Amp
2	0	0	+ 1	0
3	0	0	0	0
4	+ 2	0	+ 1	0
5	0	0	0	0
6	0	0	0	0
7	+ 2	0	+ 2	0
8	0	0	0	0
9	+ 1	Amp	+ 1	Amp
10	0	0	0	0
11	0	0	0	0
12	0	0	0	0
13	+ 1	0	+ 2	0
14	0	0	0	0
15	+ 2	0	+ 3	Amp
16	0	0	0	0
17	0	0	0	0
18	0	0	0	0
19	+ 3	Amp	+ 2	Amp
20	0	0	0	0
21	0	0	0	0
22	+ 2	0	+ 2	0
23	0	0	0	0
24	+ 2	Amp	+ 3	Amp
25	0	0	0	0
26	+ 1	0	+ 1	0
27	0	0	0	0
28	0	0	0	0
29	+ 3	Amp	+ 3	Amp
30	0	0	0	0
31	0	0	0	0
32	+ 1	0	+ 1	0
33	0	0	0	0
34	0	0	0	0
35	+ 2	Amp	+ 2	Amp
36	0	0	0	0
37	+ 1	0	+ 1	0
38	0	0	0	0
39	+ 3	Amp	+ 3	Amp
40	0	0	0	0

The median age of patients was similar between HER2-positive and HER2-negative groups (72.6 vs. 69.6 years; *p* = 0.155, Mann–Whitney U test). No statistically significant association was observed between HER2 status and ER or PR positivity (ER: *p* = 1.00; PR: *p* = 1.00, Fisher’s exact test). Likewise, the presence of lymphovascular invasion (LVI) did not differ significantly between groups (*p* = 0.13, chi-square test) (Table 2).

**Table 2:** Clinicopathologic characteristics stratified by HER2 status

Variable	HER2 Negative (*n* = 32)	HER2 Positive (*n* = 8)	*p*-value
Age, median (years)	70	74	0.19¹
Ki-67, median (%)	67	77	< 0.001¹
ER positivity, n (%)	12 (37.5%)	2 (25.0%)	1.00²
PR positivity, n (%)	5 (15.6%)	1 (12.5%)	1.00²
LVI positivity, n (%)	12 (37.5%)	6 (75.0%)	0.13²
FIGO Stage, n (%)			0.045²
– Stage I	2	0	
– Stage II	3	0	
– Stage III	15	2	
– Stage IV	12	6	

Mann–Whitney U test was used for continuous variables; chi-square test was used for categorical variables

**Fig. 1:**
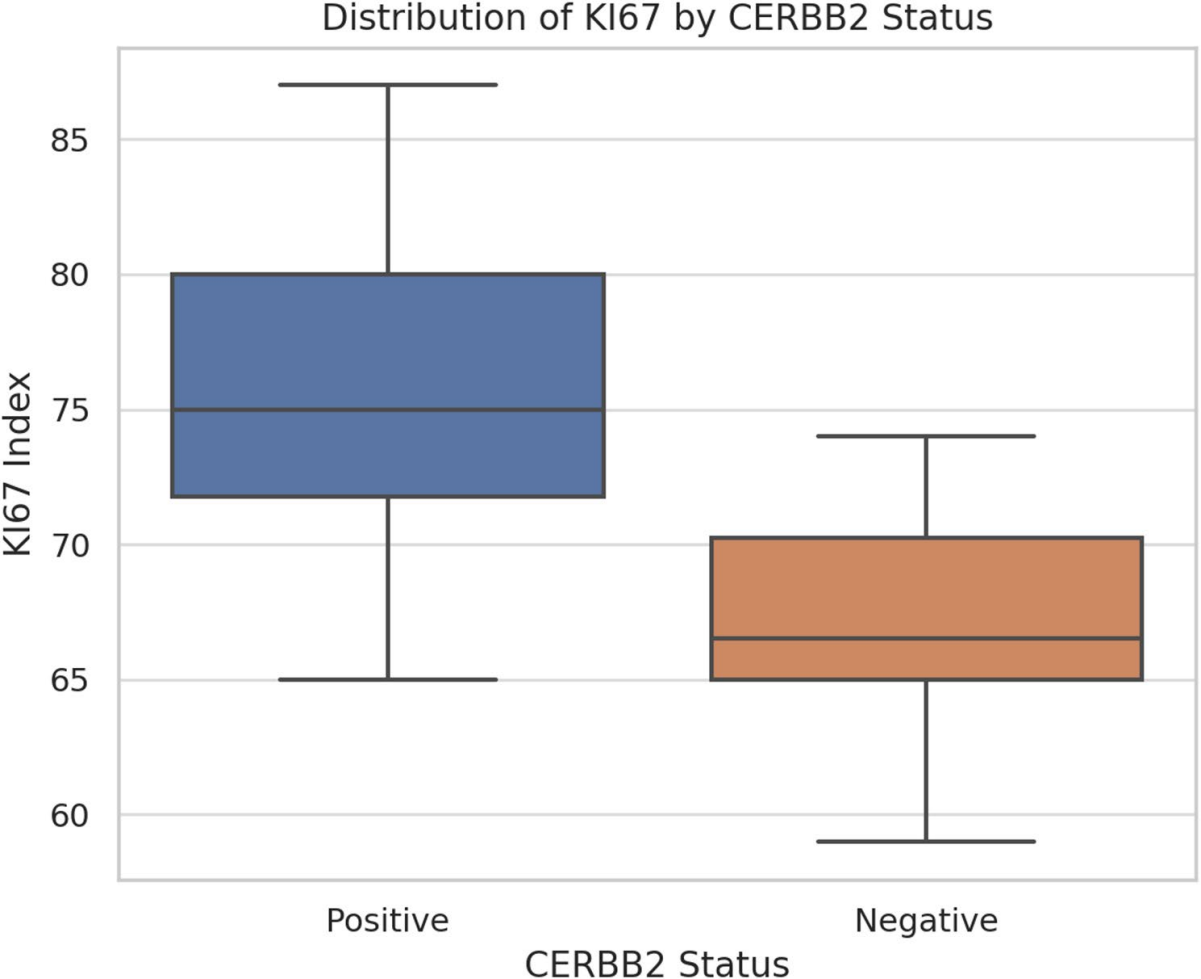
Distribution of KI67 by CERBB2 Status

In contrast, HER2 status showed significant correlations with proliferative activity and tumor stage. The Ki-67 proliferation index was significantly higher in HER2-positive tumors compared with HER2-negative cases (median 77% vs. 67%; *p* = 0.0002, Mann–Whitney U test), indicating a more aggressive biological profile (Fig. 1). FIGO stage distribution also differed significantly between groups (*p* = 0.045, Fisher’s exact test), with HER2-positive tumors more frequently presenting at advanced stages, particularly FIGO stage IV.

**Fig. 2:**
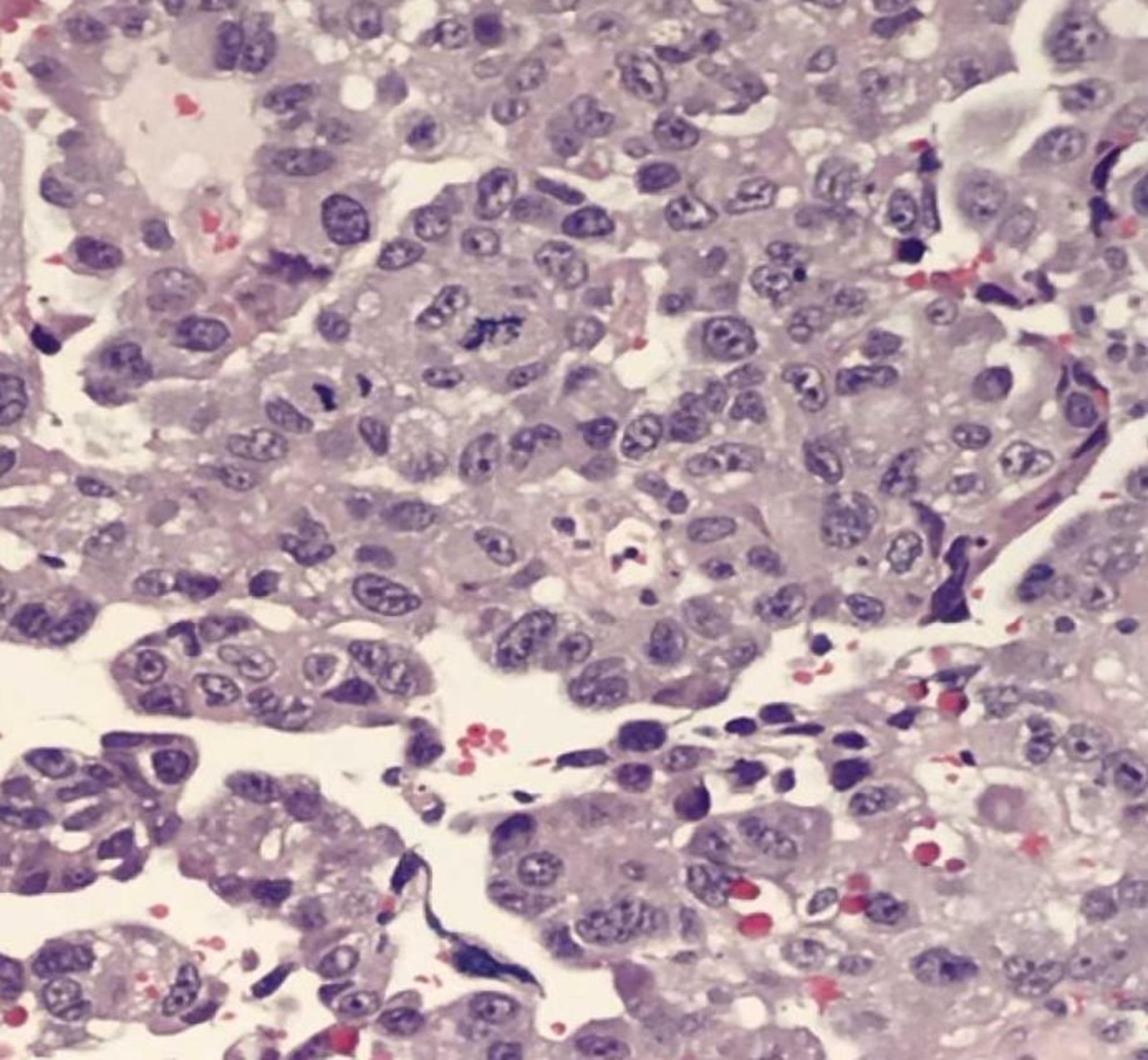
Histopathological examination at high magnification showing serous endometrial carcinoma with papillary architecture and marked nuclear atypia (H&E stain)

**Fig. 3:**
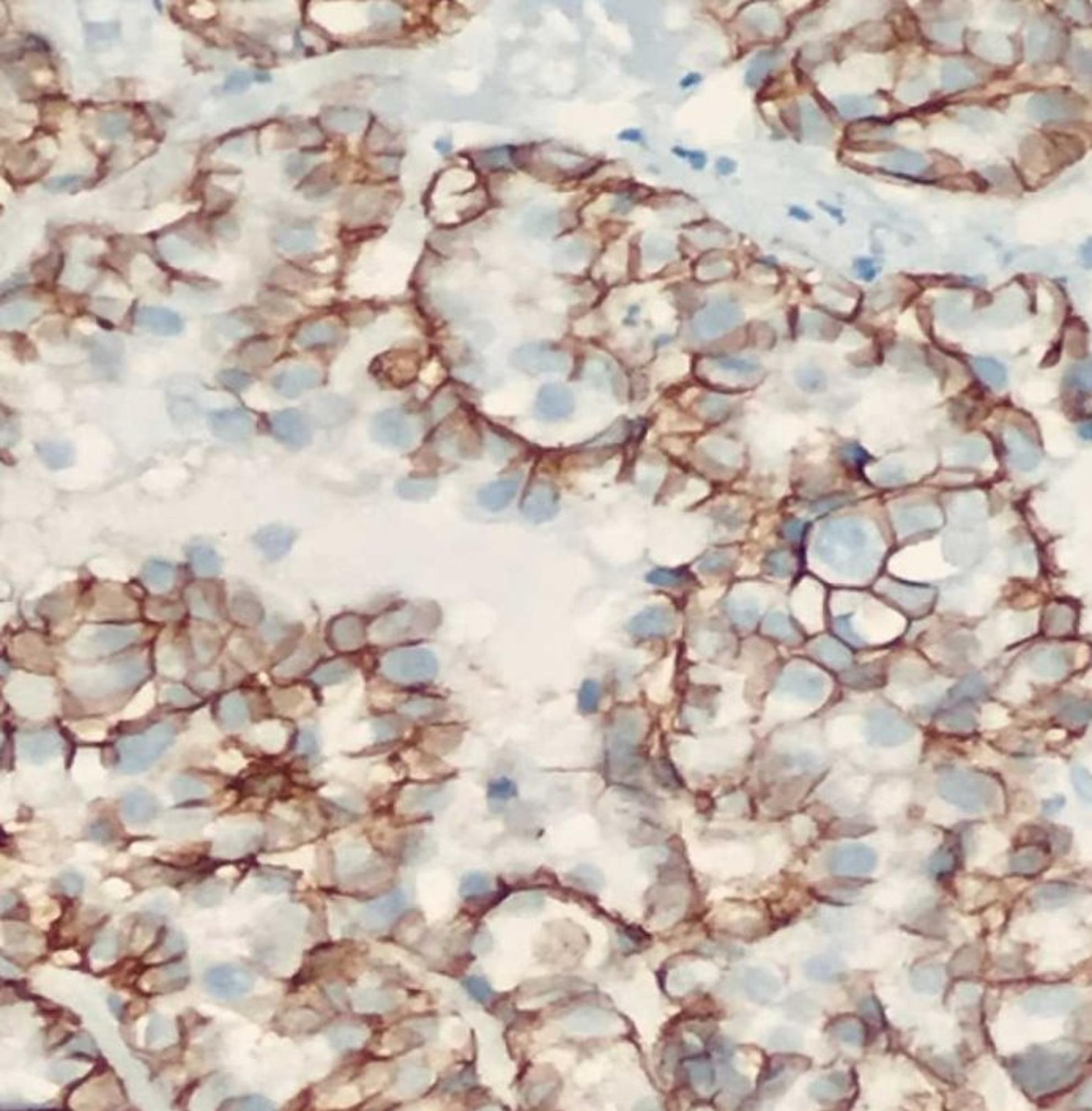
Immunohistochemical staining showing HER2 overexpression with a characteristic U-shaped membranous pattern in serous endometrial carcinoma.”

**Fig. 4:**
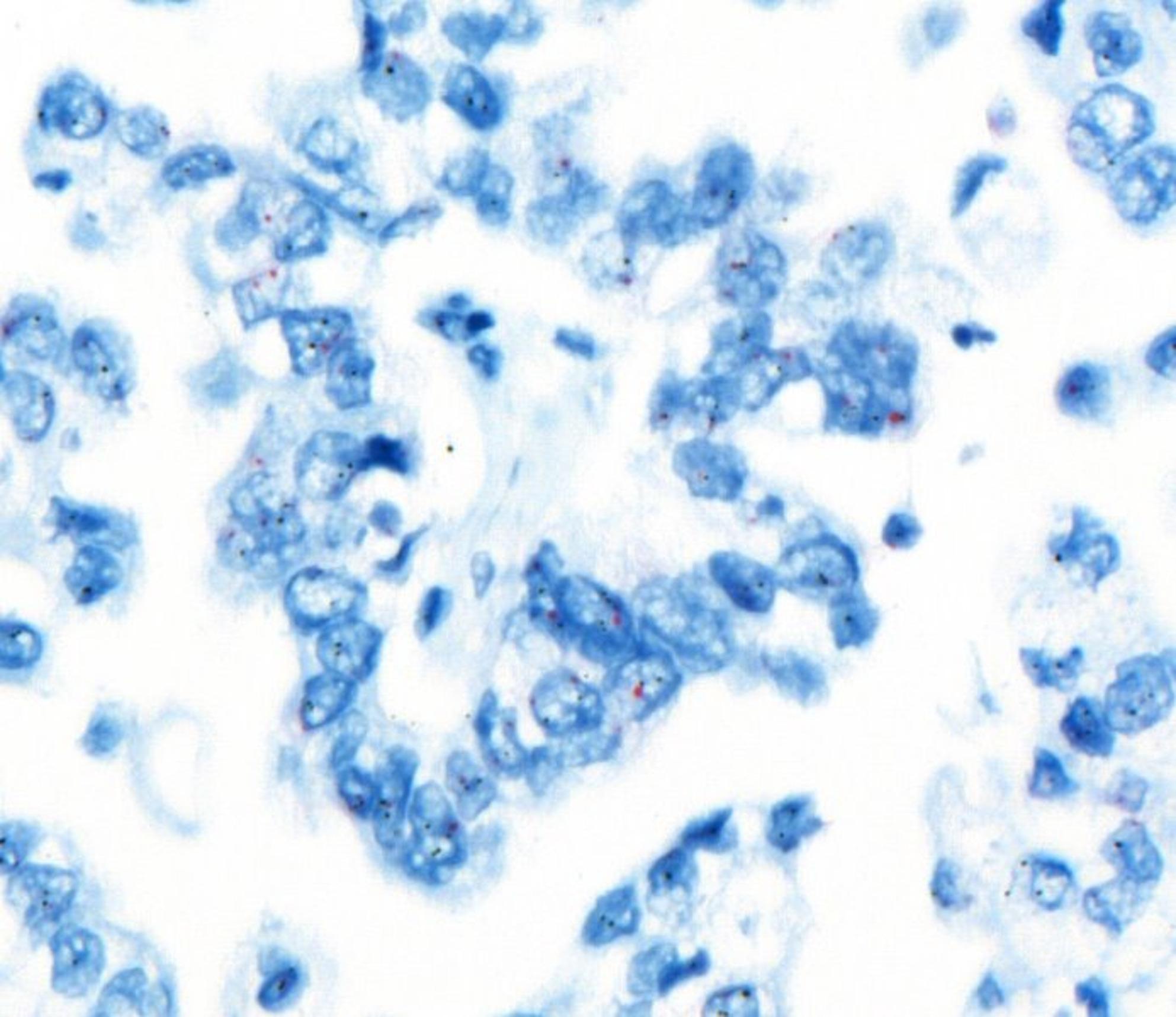
In situ hybridization (ISH) analysis showing no evidence of HER2 gene amplification in serous endometrial carcinoma

**Fig. 5:**
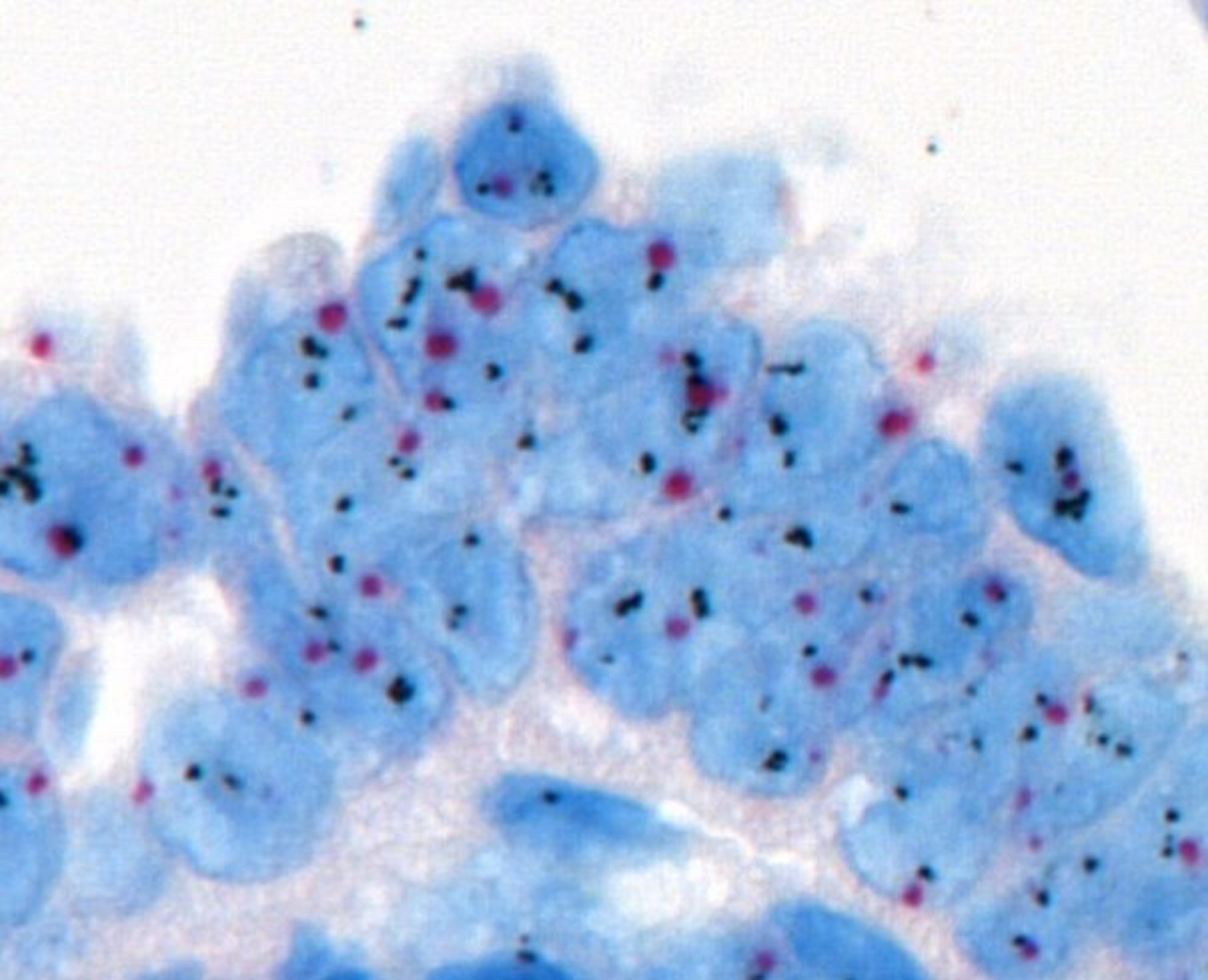
In situ hybridization (ISH) analysis demonstrating HER2 gene amplification in serous endometrial carcinoma

Histopathologically, tumors demonstrated papillary, glandular, or micropapillary architecture with pronounced nuclear atypia and pleomorphism (Fig. 2). Strong complete membranous HER2 expression (3+) was observed in a subset of cases (Fig. 3). CISH analysis confirmed HER2 gene amplification in these cases and identified additional amplification events in borderline IHC categories (Figs. 4 and 5).

Collectively, these findings indicate that HER2 amplification in uterine serous carcinoma is reliably detected in preoperative biopsy specimens, with high diagnostic accuracy and excellent concordance with resection samples. HER2-positive tumors in this cohort were also associated with higher proliferative indices and more advanced disease at presentation.

## Discussion

Uterine serous carcinoma (USC) represents one of the most aggressive histologic variants of endometrial carcinoma and accounts for a disproportionate number of cancer-related deaths despite constituting only a minority of cases [1–3, 11–14]. Its clinical behavior parallels that of high-grade serous ovarian carcinoma, with early extrauterine spread, high recurrence rates, and poor response to conventional chemotherapy. These features highlight a pressing need for reliable biomarkers that can guide tailored systemic therapies. Over the past decade, HER2 has emerged as a critical prognostic and predictive biomarker in USC, supported by compelling clinical trial evidence demonstrating improved outcomes when HER2-targeted agents—particularly trastuzumab—are combined with platinum-based chemotherapy [15]. Consequently, HER2 testing has become an essential component of the diagnostic workup for USC and is endorsed by major clinical guidelines, including the NCCN [16].

In our cohort, HER2 amplification was identified in 20% of cases based on CISH analysis of resection specimens. This rate aligns with several published series but is lower than the 30–35% HER2 positivity reported in many European and North American investigations [8–10, 12–14]. Multiple factors may explain this discrepancy, including smaller sample size, institutional differences in fixation and processing, population heterogeneity, and the inherently subjective nature of HER2 interpretation. Importantly, the lower positivity rate may also reflect our strict exclusion of inadequately fixed or heterogeneous specimens, which likely enriched our final cohort for well-preserved tissue samples. European studies have emphasized the significant inter-laboratory variability in HER2 assessment and strongly advocate for USC-specific scoring systems distinct from breast cancer criteria [8]. The population-based study from Finland further demonstrated that HER2 amplification clusters predominantly within p53-abnormal/serous-type carcinomas, confirming that HER2-driven USC represents a biologically distinct molecular subgroup [9]. Collectively, these findings underscore the importance of harmonizing HER2 testing across institutions to ensure appropriate treatment allocation.

A central strength of the present study is the simultaneous evaluation of HER2 status in paired biopsy and resection specimens. The almost perfect agreement (κ = 0.918) observed between biopsy and resection HER2 CISH results confirms that preoperative biopsies provide highly reliable information for therapeutic planning. Previous multi-institutional cohorts similarly reported high concordance rates, particularly when standardized ASCO/CAP protocols were followed and reflex ISH testing was routinely performed [12, 17]. Nonetheless, literature also documents significant discordance in 10–40% of cases, often attributable to intratumoral heterogeneity, suboptimal fixation, or sampling limitations in small biopsies [18–20]. In our study, only one false-negative biopsy was identified among eight amplified cases, yielding an NPV of 97%. This single missed case likely reflects the well-documented challenge of sampling error in tumors with patchy HER2 expression, reinforcing the need to interpret negative biopsy findings cautiously, especially in tumors with equivocal (1 + or 2+) IHC patterns.

Intratumoral heterogeneity remains one of the defining challenges of HER2 assessment in USC. Unlike the uniform circumferential membranous staining characteristic of HER2-positive breast carcinomas, USC often exhibits incomplete, basolateral, or mosaic staining patterns, making interpretation considerably more difficult [12]. Amplification may also be regionally restricted, as illustrated by studies documenting 13–97% heterogeneity across tumor Sects [18, 20]. In this context, our finding of isolated amplification in one IHC 1 + case and three IHC 2 + cases is particularly meaningful. It reinforces the principle that IHC alone cannot reliably identify all HER2-amplified USC cases, especially those with low or equivocal expression. Consequently, reflex CISH or FISH testing is indispensable not only in 2 + cases but also in selected 1 + tumors when morphology and clinical context raise suspicion for USC. These observations align with prior reports by Buza et al. and Banet et al., who demonstrated that a subset of HER2-low tumors may harbor clinically relevant amplification [12, 20].

The strong correlation observed between HER2 positivity, elevated Ki-67 proliferation index, and advanced FIGO stage further highlights the biological aggressiveness of HER2-driven USC. Our results are consistent with prior evidence showing that HER2-amplified tumors tend to exhibit higher proliferative activity, increased genomic instability, and more extensive disease at diagnosis [12–14]. The absence of association between HER2 status and ER/PR expression also reflects the characteristic hormone receptor–negative profile of serous carcinomas and is in line with molecular classification frameworks in which HER2-positive USC belongs to the p53-abnormal subgroup rather than the hormone-driven endometrioid spectrum [21].

An emerging area of investigation relevant to our findings involves the concept of “HER2-low” tumors. Although extensively characterized in breast cancer, HER2-low endometrial carcinomas are poorly defined, and their clinical relevance remains uncertain. The presence of gene amplification in a single IHC 1 + tumor in our cohort adds to growing evidence that HER2-low USC may harbor clinically significant alterations and may benefit from novel HER2-directed therapies such as antibody–drug conjugates. As therapeutic landscapes evolve, there is a compelling need to better characterize HER2-low USC and determine its responsiveness to emerging HER2-targeted agents.

Our results also emphasize the importance of preanalytic standardization. By excluding specimens with inadequate fixation, variable biopsy techniques, or consultation materials, we ensured high tissue quality and minimized technical artifacts that could compromise HER2 interpretation. This strategy likely contributed to the high biopsy–resection concordance observed, supporting standardized tissue processing as an essential prerequisite for accurate HER2 assessment. However, it also reduced our sample size and may limit the generalizability of the findings.

In summary, our study demonstrates that biopsy-derived HER2 testing is highly reliable for predicting HER2 status in USC and supports the integration of HER2 analysis into preoperative diagnostic workflows. The observed correlations between HER2 positivity, tumor proliferation, and advanced stage reinforce the prognostic and therapeutic significance of HER2 in this aggressive malignancy. Our findings further highlight the necessity of dual IHC/ISH testing, particularly in low-level or equivocal cases, and contribute to the ongoing movement toward USC-specific HER2 scoring criteria and harmonized testing protocols. As HER2-targeted therapies—particularly those effective in HER2-low disease—continue to expand, accurate and reproducible HER2 evaluation will remain central to optimizing patient outcomes.

## Conclusion

HER2 plays a critical prognostic and therapeutic role in uterine serous carcinoma, a biologically aggressive and clinically challenging subtype of endometrial cancer. In this study, we demonstrated that HER2 assessment in endometrial biopsy specimens shows *almost perfect* concordance with resection materials, supported by a high kappa coefficient (κ = 0.918), a positive predictive value of 100%, and a negative predictive value of 97%. These findings confirm that preoperative biopsy-based HER2 testing is highly reliable and can be confidently used to guide treatment decisions, including the selection of patients for HER2-targeted therapy.

Our results further show that HER2 amplification is significantly associated with higher FIGO stage and increased proliferative activity (Ki-67), underscoring the biological aggressiveness of HER2-positive USC. The identification of amplification in both equivocal (2+) and low-level (1+) IHC cases highlights the essential role of reflex CISH testing, particularly in USC where intratumoral heterogeneity is common.

Collectively, our findings support a dual IHC/ISH testing approach and reinforce the need for USC-specific HER2 scoring guidelines. Standardized pre-analytic tissue handling is crucial to ensure diagnostic accuracy, and biopsy-based HER2 evaluation should be integrated into routine preoperative assessment. Future studies with larger, multicenter cohorts are warranted to validate HER2-low classifications, investigate their therapeutic relevance, and explore the impact of HER2 status on treatment response and survival outcomes.

## Limitations

One of the major limitations of this study is the substantial number of cases excluded due to pre-analytical variables. Specimens with inadequate fixation quality, those obtained through heterogeneous biopsy techniques, or samples submitted as external consultations were eliminated to preserve methodological consistency. Although these exclusion criteria were necessary to ensure the most accurate assessment of biopsy–resection concordance, they inevitably reduced the sample size and, to some extent, limited the statistical power of the study. Additionally, because this investigation focused exclusively on diagnostic concordance, it did not evaluate the relationship between HER2 status and clinical outcomes such as survival or treatment response. Therefore, the prognostic and predictive implications of HER2 expression in this cohort could not be fully explored.

## Data Availability

The datasets generated and/or analyzed during the current study are available from the corresponding author on reasonable request.

## Abbreviations

USC: Uterine serous carcinoma
IHC: Immunohistochemistry
CISH: Chromogenic in situ hybridization
HER2: Human epidermal growth factor receptor 2
PPV: Positive predictive value
NPV: Negative predictive value
PFS: Progression-free survival
OS: Overall survival

## References

[1] Surveillance, Epidemiology and End Results Program. Cancer stat facts: uterine cancer. National Cancer Institute. https://seer.cancer.gov/statfacts/html/corp.html. Accessed 28 Jan 2023.

[2] Matthews RP, Hutchinson-Colas J, Maiman M, et al. Papillary serous and clear cell type lead to poor prognosis of endometrial carcinoma in black women. Gynecol Oncol. 1997;65(2):206–12. 10.1006/gyno.1997.4617. 9159326 10.1006/gyno.1997.4617

[3] Hamilton CA, Cheung MK, Osann K, et al. Uterine papillary serous and clear cell carcinomas predict for poorer survival compared to grade 3 endometrioid corpus cancers. Br J Cancer. 2006;94(5):642–6. 10.1038/sj.bjc.6603012. 16495918 10.1038/sj.bjc.6603012 PMC2361201

[4] Talia KL, Banet N, Buza N. The role of HER2 as a therapeutic biomarker in gynaecological malignancy: potential for use beyond uterine serous carcinoma. Pathology. 2023;55(1):8–18. 36503635 10.1016/j.pathol.2022.11.004

[5] Buza N. HER2 testing and reporting in endometrial serous carcinoma: practical recommendations for HER2 immunohistochemistry and fluorescent in situ hybridization: proceedings of the ISGyP companion society session at the 2020 USCAP annual meeting. Int J Gynecol Pathol. 2021;40(1):17–23. 10.1097/PGP.0000000000000711. 33290351 10.1097/PGP.0000000000000711

[6] Wolff AC, Hammond ME, Schwartz JN, et al. American society of clinical Oncology/College of American pathologists guideline recommendations for human epidermal growth factor receptor 2 testing in breast cancer. Arch Pathol Lab Med. 2007;131(1):18–43. 19548375 10.5858/2007-131-18-ASOCCO

[7] Wolff AC, Hammond MEH, Allison KH, et al. Human epidermal growth factor receptor 2 testing in breast cancer: ASCO/CAP clinical practice guideline focused update. Arch Pathol Lab Med. 2018;142(11):1364–82. 29846104 10.5858/arpa.2018-0902-SA

[8] Hodgson A, Parra-Herran C, Yemelyanova A, et al. Reproducibility of scoring criteria for HER2 immunohistochemistry in endometrial serous carcinoma: a multi-institutional interobserver agreement study. Mod Pathol. 2021;34(7):1274–83. 10.1038/s41379-021-00761-2. 10.1038/s41379-021-00746-5 33536574

[9] Aro K, Pasanen A, Leminen A, Loukovaara M, Bützow R. HER2 amplification and HER2-low expression in endometrial carcinoma: a population-based study. BJC Rep. 2025;4:100163. 10.1038/s44276-025-00125-6. 10.1038/s44276-025-00125-6 PMC11821901 39939712

[10] Odicino FE, Bignotti E, Rossi E, et al. HER2 overexpression and amplification in uterine serous papillary carcinoma: correlation with clinicopathological features and outcome. Gynecol Oncol. 2025;178:78–85. 10.1016/j.ygyno.2024.12.012.

[11] McGunigal M, Liu J, Kalir T, Chadha M, Gupta V. Survival differences among uterine papillary serous, clear cell and grade 3 endometrioid adenocarcinoma: a National cancer database analysis. Int J Gynecol Cancer. 2017;27(1):85–92. 10.1097/IGC.0000000000000844. 27759595 10.1097/IGC.0000000000000844

[12] Buza N, English DP, Santin AD, Hui P. Toward standard HER2 testing of endometrial serous carcinoma: 4-year experience at a large academic center and recommendations for clinical practice. Mod Pathol. 2013;26(12):1605–12. 10.1038/modpathol.2013.113. 23765245 10.1038/modpathol.2013.113

[13] Erickson BK, Najjar O, Damast S, et al. Human epidermal growth factor receptor 2 in early-stage uterine serous carcinoma: a multi-institutional cohort study. Gynecol Oncol. 2020;159(1):17–22. 10.1016/j.ygyno.2020.07.016. 32709539 10.1016/j.ygyno.2020.07.016 PMC7541557

[14] Santin AD, Bellone S, Siegel ER, et al. Racial differences in the overexpression of HER2/neu: a major prognostic indicator in uterine serous papillary cancer. Am J Obstet Gynecol. 2005;192(3):813–8. 10.1016/j.ajog.2004.10.605. 15746676 10.1016/j.ajog.2004.10.605

[15] Fader AN, Roque DM, Siegel E, et al. Randomized phase II trial of carboplatin–paclitaxel versus carboplatin–paclitaxel–trastuzumab in uterine serous carcinomas that overexpress HER2/neu. J Clin Oncol. 2018;36(20):2044–51. 10.1200/JCO.2017.76.5966. 29584549 10.1200/JCO.2017.76.5966

[16] National Comprehensive Cancer Network. Uterine Neoplasms. Version 4.2019. https://www.nccn.org/professionals/physician_gls/#site. Accessed 6 Oct 2019.

[17] Slomovitz BM, Broaddus R, Burke TW, et al. HER2/neu overexpression and amplification in uterine serous carcinoma. Gynecol Oncol. 2020;159(3):744–51. (Sayfalar eksikse lütfen gönder, tamamlayayım.).33019982

[18] Buza N, Hui P. Marked heterogeneity of HER2/neu gene amplification in endometrial serous carcinoma. Genes Chromosomes Cancer. 2013;52(12):1178–86. 24123408 10.1002/gcc.22113

[19] Halle MK, Tangen IL, Berg HF, et al. HER2 expression patterns in paired primary and metastatic endometrial cancer lesions. Br J Cancer. 2018;118:378–87. 29169184 10.1038/bjc.2017.422 PMC5808034

[20] Banet N, Shahi M, Batista D, et al. HER2 amplification in uterine serous carcinoma and serous endometrial intraepithelial carcinoma. Am J Surg Pathol. 2021;45(5):708–15. 33739786 10.1097/PAS.0000000000001682

[21] Köbel M, Rahimi K, Rambau PF, et al. An immunohistochemical algorithm for molecular classification of endometrial carcinoma: a provisional strategy. Mod Pathol. 2018;31(3):452–62.29052601

